# A Smartphone-Gamified Virtual Reality Exposure Therapy Augmented With Biofeedback for Ailurophobia: Development and Evaluation Study

**DOI:** 10.2196/34535

**Published:** 2024-03-06

**Authors:** Ali Khaleghi, Abbas Narimani, Zahra Aghaei, Anahita Khorrami Banaraki, Peyman Hassani-Abharian

**Affiliations:** 1 Iranian Light Source Facility Institute for Research in Fundamental Sciences Tehran Iran; 2 Department of Computer Engineering Imam Khomeini International University Qazvin Iran; 3 Department of Computer Engineering Bu-Ali Sina University Hamedan Iran; 4 Brain and Cognition Clinic Institute for Cognitive Science Studies Tehran Iran; 5 Department of Cognitive Psychology and Rehabilitation Institute for Cognitive Science Studies Tehran Iran

**Keywords:** animal phobia, specific phobia, ailurophobia, cat phobia, biofeedback, smartphones, virtual reality, gamification, mobile phone

## Abstract

**Background:**

To the best of our knowledge, no specialized research has been conducted to address ailurophobia (fear of cats) in Iran or globally. This has driven our project, along with the prevalence of ailurophobia and the absence of a gamified virtual reality exposure therapy (VRET) that incorporates affordable and easily accessible biofeedback (BF) tools. We hypothesize that a gamified VRET augmented with BF will yield more positive effects than a similar device lacking BF.

**Objective:**

This study primarily focuses on the development and preliminary evaluation of a smartphone-gamified VRET integrated with BF, targeting animal phobia, with a specific case study on ailurophobia. The secondary objectives are using affordable and readily available BF found in devices such as smart bands and smartwatches and creating a mobile virtual reality gamified app to improve patients’ adherence to treatments while simultaneously enhancing the app’s accessibility, scalability, and outreach.

**Methods:**

Evaluations encompassed 3 methods. First, we identified the tool’s potential positive effects on phobia interventions, exploring 4 effects: intrinsic motivation, simulation of fearful situations, management of stressful circumstances without therapists’ presence and mitigation of catastrophic thoughts, and preliminary effects on ailurophobia treatment. Participants were divided into BF and non-BF groups. Second, we gathered user preferences and opinions about the treatment. Third, we conducted heuristic evaluations using 44 heuristics from existing system usability scales assessing user interfaces, virtual reality platforms, and video games’ playability. To interpret the data, mean scores; ANOVA, single factor; and ANOVA, 2-factor with replication were used. A total of 29 individuals were identified, of which 10 met the eligibility criteria or were accessible.

**Results:**

The smartphone-gamified VRET augmented with BF exhibited better results on the identified effects compared with the non-BF version and contributed to normalizing encounters with cats. Moreover, 41 of the 44 heuristics achieved a percentage above 62%, indicating its potential as a therapeutic product and its ability to enhance patient adherence to treatments. Patient preferences on the treatment and its strengths and weaknesses were provided for further improvement.

**Conclusions:**

The tool has the potential to evolve into a comprehensive solution by incorporating various types of cats and their behaviors, simulating environments in which they are commonly found, and enhancing its appeal through an increased sense of adventure without inducing unrealistic fears. By adapting fear elements, the game can be tailored to treat various animal phobias. Phobia-focused games should avoid action and combat scenarios to prevent reinforcement of fear responses. After rigorous evaluation, further exploration is required to provide remote use beyond clinical settings.

## Introduction

### Specific Phobia and Available Therapies

Specific phobia is the most common anxiety disorder, with a lifetime prevalence of 12.5% [1]. It is characterized by an extreme and persistent fear of a specific object or situation [2], leading to substantial disruptions in daily life and heightened anxiety. Many individuals restructure their lives to evade their fears over extended periods [3,4]. Prolonged phobia detrimentally affects academic, social, and family aspects, compromising overall quality of life [5]. Situational (eg, fear of enclosed spaces and flying), natural environment (eg, fear of heights and storms), animal (eg, fears of snakes, spiders, and cats), and blood or injection or injury (eg, fears of medical procedures and seeing blood) fears are subtypes of specific phobias, with animal and natural environment phobias being more prevalent [3].

Phobia interventions are categorized into exposure therapies (eg, direct in vivo exposure, systematic desensitization, imaginal exposure, and virtual reality [VR]) and nonexposure approaches (eg, cognitive therapy and progressive muscle relaxation). There has been a trend toward adopting brief, intensive, or concentrated treatments to manage anxiety [5]. Among the available treatments, exposure therapies are the most commonly used approach for specific phobias [6]. However, although specific phobias are highly treatable, only 31% of patients seek treatment and, among those, only 43.4% seek mental health services [3]. Moreover, some patients might be unable to complete the treatment because of severe reactions, resulting in an attrition rate of 45% [3,7]. In total, 3 main factors contribute to this percentage [7]: (1) perceiving treatments as highly aversive and frightening; (2) the need to visit clinics throughout the treatment, causing relationship and ethical issues, such as perceived cruelty when therapists intentionally evoke fear; and (3) the lack of appealing treatments.

### Gamified VR Exposure Therapies Augmented With Biofeedback

To overcome the limitations of exposure therapy methods, incorporating new technologies becomes imperative. Gamification, VR, and biofeedback (BF) are promising options. However, our research indicates that few studies have simultaneously used these technologies for specific phobias. Virtual reality exposure therapy (VRET) uses 360° computer-generated simulations [8,9] similar to traditional exposure therapies [2]. Meta-analyses have shown that VRETs are effective and their performance can rival standard exposure therapies [2]. VR’s application in cognitive impairment, anxiety disorders, pain management, phobias, posttraumatic stress disorder, rehabilitation, and eating disorders, among others, has surged because of its immersive realism [8,10]. To treat phobias, VR is a safer, less embarrassing, and cost-effective solution by simulating fear-inducing situations in a controlled environment [8,9,11]. However, VR alone may not address all exposure therapy disadvantages, and enhancing the attractiveness of VRETs is crucial for treatment success. Researchers have explored the potential of gamified VRETs in treating phobias [2,12,13]. Gamification, a strategy derived from video game–based approaches, has proven successful in achieving serious objectives across various fields, including the workplace [14], education [15], marketing [16], mental health [17-19], learning disabilities [20,21], and lazy eye treatment [22]. The primary inherent feature of digital games is their high-level motivational potential [23]. Video games’ appeal, engagement, and effectiveness encourage players and frequent use [18]. Attractiveness is beneficial for overcoming people’s reluctance to seek treatment, broadening the reach of gamified interventions [18]. The engaging nature of gamification enhances users’ experiences, as players are driven to win, explore stories, and ultimately reduce attrition rates [12,18,24]. The effectiveness aspect offers opportunities for achieving serious objectives such as behavior changes [18]. In a gamified product, elements such as scores, badges, and levels are integrated from games into nongame contexts, while not necessarily offering a complete gaming experience [18,25].

Human emotion recognition sensors or BF is another technology that can enhance gamified interventions. This technology serves 2 crucial purposes. First, it boosts their level of attractiveness by leveraging a strategy commonly used in video games to increase engagement [26]. Second, it addresses some of the limitations of traditional methods by potentially reducing or eliminating the need for therapists’ constant presence. These sensors work by measuring various body parameters or electrical impulses in the nervous system to identify different emotions and track their changes [27]. Common techniques include electroencephalography, skin resistance measurements, blood pressure, heart rate (HR), eye activity, and motion analysis. With advancements in chipset manufacturing, BF has become more accessible, portable, efficient, and affordable. Users can easily access their data, thereby enabling self-regulation and monitoring. These technologies are incorporated into smart wristbands and watches to help individuals regulate anxiety in their daily lives. BF therapies have shown positive effects in treating conditions such as migraines [28] and attention-deficit/hyperactivity disorder in children [29].

### Objectives

The primary aim of this study was to develop and conduct a preliminary assessment of smartphone-gamified VRET augmented with BF for the treatment of cat phobia (ailurophobia). We hypothesize that this tool will outperform gamified VRET without BF in various aspects. Limited evidence exists on animal phobia in Iran, particularly ailurophobia. Observations at the Cognitive and Brain Clinic in Tehran revealed a substantial prevalence of this phobia, as reported by the fourth author, who is a cognitive expert and psychologist attending to cat phobia patients daily. Owing to the abundance of cats in most Iranian cities, encounters are inevitable, resulting in daily challenges for patients walking on the streets and alleys. The secondary objectives were as follows:

1. Using affordable and accessible BF tools in devices such as smart bands and smartwatches to serve as both BF and a game mechanic, enhancing engagement and efficacy.
2. Developing a mobile VR–gamified app to enhance patients’ adherence to phobia treatment and expand the app’s accessibility, scale, and reach.

To evaluate the effectiveness of the tool, its potential positive effects on phobia interventions were examined. The tool’s impact on the effects was examined by dividing the participants into BF and non-BF groups. In addition, we considered the playability and usability aspects of the tool, along with patients’ preferences, to optimize its performance and enhance usability for future improvements.

## Methods

### Design and Development

**Figure 1:**
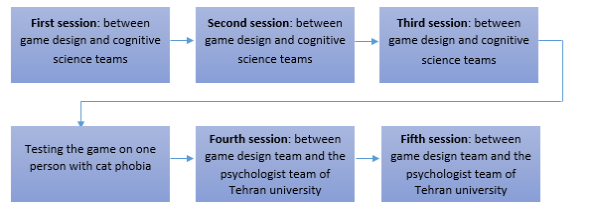
The overall view of sessions between the game designers and other related experts.

Our primary objective was to present fear elements indirectly to the player, ensuring that interacting or not interacting with them would not affect the game’s progress. The secondary objective was to create a general game design model that could be easily customized for specific phobias, particularly animal phobias. During the initial game development meeting, 2 game design experts (a game designer and a gamification expert) collaborated with 2 cognitive science experts (one of whom also specialized in cognitive games). They engaged in a discussion regarding the essential components required to simulate stress. Size, color, and behavior of the stimuli were introduced as fundamental elements for replicating the desired scenarios. The game team then devised the game stages using a maze design. In the second meeting, cognitive experts suggested simplifying the design to accommodate players of all ages. As it involved memory and problem-solving, it was rejected, leading to a more straightforward game plan that focused on finding lost objects in a park. In the third session, this plan received approval and was tested on a woman aged 40 years with cat phobia, who was selected based on her self-reported fear of cats. She stated, “I experienced a lot of fear during playing.” In the fourth session, minor adjustments were made to the game. In total, 2 psychologists from Tehran University found the initial voice of the guide annoying, thereby hindering patient motivation. The overall view of the sessions is presented in Figure 1.

Our game’s storyline was inspired by the “Hot and Cold” game. One group hides an object, and the other group should find it using verbal clues such as “colder” as they move away and “hotter” as they get closer. The experience is similar to that of a park with diverse paths. Players are on a quest to discover diamonds concealed within treasure boxes, all while walking along these pathways. Each game session comprises 4 short yet consecutive levels. At each level, players must determine their distance from each box by perceiving changes in the sound consistently played. Moreover, a hint ribbon shows the player’s distance to the box for increased engagement. After locating the box, players must stay in front of it for a specific duration to open it, with the time increasing at later levels. The players must open the previous level’s box to unlock the next challenge.

Regarding authors’ concerns about spreading the game to individuals with phobias, smartphones were chosen as the primary platform. Using smartphones as a VR tool requires affordable mobile VR glasses, which are significantly cheaper than other options such as Oculus or HTC VR. The primary challenge in mobile VR is the user interaction limitations. The game uses Gaze, a pointer on the screen that allows users to interact through head movements, thus providing a mouselike experience. In addition, the game incorporates joysticks connected to the phone, thus offering more interactive possibilities.

The intensity of the fear elements must be balanced based on the game’s progress and levels, as in previous studies [7,13]. The escalation of fear stimuli is determined by the following features, each with its own difficulty level. Moreover, these elements can be further amplified in tandem with player’s advancement.

**Figure 2:**
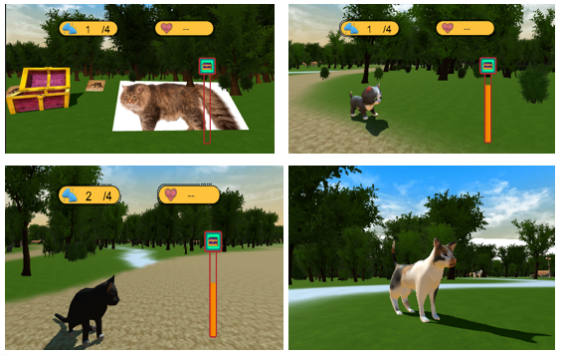
Screenshots of the game’s environment with all its cat types.

1. Visual elements: the fear-triggering elements include cat photos, fantasy cat models, low-poly cat models with minimal details, and high-poly cat models that closely resemble real cats. According to experts, individuals who fear something may also react to objects and shapes that resemble it. For instance, people who are afraid of cats might experience fear when encountering a cat picture or a furry object. This phenomenon is directly related to the degree and intensity of the individual’s fear [30]. Figure 2 illustrates the game environment.

2. Fear elements’ sound: the scary elements vary from silent to those with terrifying sounds. In intense situations, cats produce specific sounds that could heighten anxiety. The timing of when the sound is played also adds to the diversity. For example, when players are near a cat, the sounds it emits could intensify their fear.

4. The quality of fear elements’ behaviors: studying the behavior quality of a stimulus is under investigation [31,32]. A cat jumping from one point to another evokes more fear than a cat simply standing still. Various animations were designed for 3D model cats. The fantastical cat playfully turns its head and randomly spins around. The low-poly cat remains stationary, solely turning its head. In the final level, the high-poly cat features 3 different animations. The first 2 animations portrayed the cat at rest, either shaking its tail and head or cleaning its paws. The third animation involves the cats’ walking behavior.

5. Interactable elements: fear elements that respond to the player’s presence add a sense of authenticity to the game, elevating immersion and allowing for anxiety manipulation. Cats may react by turning, approaching, or fleeing when a player gets closer. Both low-poly and high-poly cats respond to the player’s presence. The manner in which the elements react was also classified. Although the fantasy cat remains unresponsive, the low-poly cat acknowledges players by turning their heads and looking at them when they enter the zone. At the last level, the realistic cat not only faces the players but also follows them until they exit from its zone.

6. Fear elements’ size: element size could also amplify fear. In the final level, some cats are larger, preparing players to confront more intimidating situations.

7. Fear elements’ numbers**:** seeing numerous cats creates a feeling of being surrounded, indirectly encouraging players to confront their fear. As players approach the boxes, fear intensifies, peaking around those areas.

**Figure 3:**
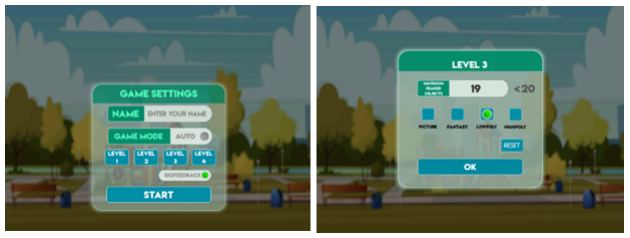
Images of the level customization menu.

The quantity and type of elements can be customized based on players’ preferences and conditions (Figure 3), which is beneficial when they need to concentrate on a specific scary element. Furthermore, a player who does not fear an element can eliminate it from the game.

A VR Android game was developed using the Unity game engine, incorporating the Amazfit Bip smartwatch. In anxiety treatments, HR variability is a common BF technique for stress management [33]. However, because of limitations in receiving these signals through conventional smartwatches and wrist bands, HR was used instead of HR variability. HR data are accessible in smartwatches through Bluetooth low energy technology [33]. A plug-in for the Unity3D game engine was implemented to integrate smartwatch data into the game. The player’s HR was incorporated into the experience as a game mechanic. The HR was displayed on the corner of the screen. A total of three BF techniques were used in this study: (1) displaying changes in players’ bodies to inform and manage anxiety [33]; (2) keeping HR within specific limits allows players to earn the game’s prize, a diamond, promoting relaxation skills for stressful situations [34]; and (3) maintaining a low HR for a period allowed players to open boxes and collect more diamonds [33,35].

### Trial Design, Participants, and Procedure

#### Overview

In total, three methods were used to evaluate the tool: (1) identifying its potential positive effects it could have in phobia interventions. To assess the game’s impact on these effects, participants were divided into BF and non-BF groups, with the only differences being the use of smartwatches; (2) gathering user preferences about the treatment; and (3) considering the tool’s playability and usability aspects for subsequent optimization and improved usability.

#### Ethical Considerations

This study was approved by the Research Ethics Committees of the Institute for Cognitive Science Studies (IR.UT. IRICSS.REC.1401.047). Informed consent was obtained from participants. They had the freedom to withdraw from the study at any time. The participants’ data were anonymized. To compensate for time, participants were informed that a smartphone-compatible version of the game would be provided free after its finalization.

#### Participants

The snowball method was used for recruitments. One attractive advertisement was prepared in Farsi and shared within various working, educational, and family groups on Instagram and WhatsApp. Receivers were asked to help by sharing the advertisement with their own groups. Recruitment took place from September 8 to October 14, 2022, in 2 provinces in Iran: Lorestan and Tehran. Each test session lasted up to 3 hours, and the participants had the flexibility to choose the test location. Random assignment was used to allocate the participants to the study arms.

**Figure 4:**
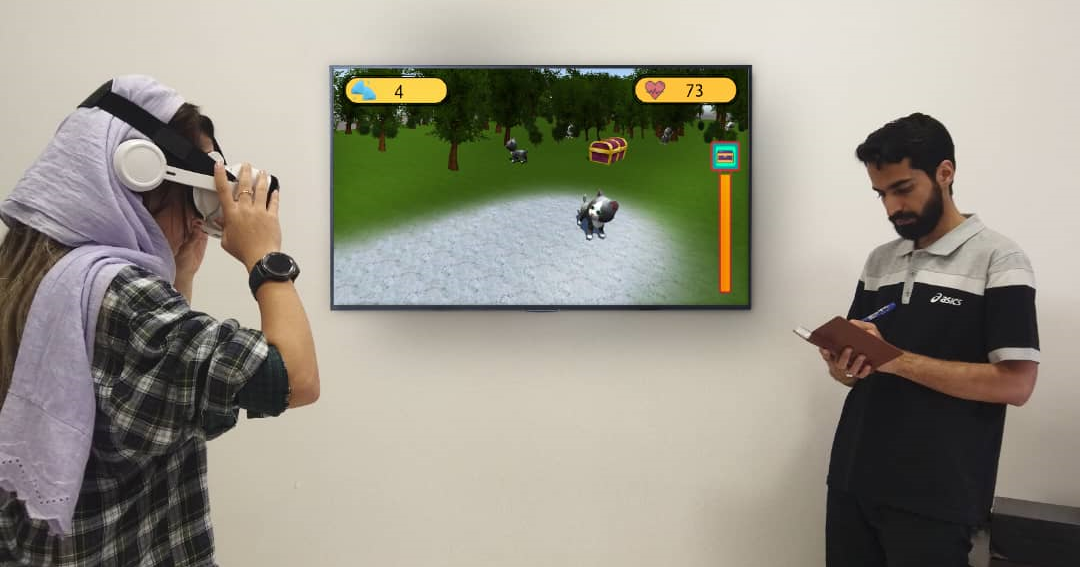
Depiction of participants.

Inclusion criteria were (1) providing informed written consent, (2) understanding and reading Persian, and (3) scoring ≥55 on the Fear of Cats Questionnaire (FCQ). Exclusion criteria were (1) currently receiving psychological treatment for ailurophobia; (2) having another severe mental disorder (alcohol or substance abuse, psychotic disorder, dementia, or bipolar disorder); (3) diagnosed with a severe personality disorder; (4) experiencing depressive symptoms or suicidal ideation; (5) heart disease; (6) vision or balance problems affecting the VR experience; (7) pregnancies exceeding 3 months; and (7) fear of cats only in a few and exceptional cases. An image of participants is presented in Figure 4.

#### Identifying the Positive Effects of the Gamified VRET Augmented With BF

##### Effect 1: Intrinsic Motivation

One primary positive effect that the app could have on phobia interventions is its ability to enhance intrinsic motivation. By incorporating gamification, VR, and BF, the app effectively motivated patients to actively engage in their treatment. We hypothesize that combining gamified VRET with BF will significantly increase motivation compared with a similar tool without BF.

To assess their impact on intrinsic motivation, we used subjective and objective measures. After each level, participants completed a 10-item questionnaire that was previously used to evaluate subjective engagement [36-39].

Participants played a minimum of 5 levels and completed the intrinsic motivation questionnaire after each level, except for the first. First-level data were excluded because of participants’ unfamiliarity with the experience. For the training step, the picture level of the game with 8 cat pictures was predetermined. The other settings regarding the type and quantity of stimuli for mandatory games are as follows:

- Game 1: fantasy model with 13 cats
- Game 2: low-poly model with 19 cats
- Games 3 and 4: high-poly model with 23 cats

We deliberately chose the last 2 steps in the same manner to examine the impact of repetitive tasks on the participants.

After the mandatory games, participants had the option to play the game for up to 4 additional times. During the voluntary sessions, participants were allowed to choose the type and number of cats, but the number of cats had to be selected in ascending order. In these sessions, we used a shorter version of the intrinsic motivation questionnaire with only 5 items, as used in Lumsden et al [36].

##### Effect 2: Simulating Fearful Situations

For phobia treatments to be effective, the game should evoke fear among individuals. To evaluate this, both groups were asked to rate their anxiety levels on a scale from 1 (“no anxiety”) to 10 (“extreme anxiety”) after any mandatory and voluntary sessions (except the first level).

##### Effect 3: Controlling Stressful Circumstances, Eliminating Therapists’ Presence, and Mitigating Catastrophic Thoughts

The game enables participants to implicitly learn relaxation techniques while confronting their fears. The box-opening mechanism involves standing in front of the box for gradually increasing durations. This combined approach, along with BF, has the potential to reduce the need for therapists’ presence. After the experiments, the participants were asked two questions: (1) How well do you think you could manage your stress when dealing with a real cat after using the gamified app? (2) To what extent can our game eliminate the need for operators? The app’s attractive and fantasy environment was expected to alleviate catastrophic thoughts. Participants were also encouraged to share any positive signs of reducing their frequency of thinking about their fears.

##### Effect 4: Preliminary Effects on Ailurophobia Treatment

The study used before and after assessments with the State-Trait Anxiety Inventory (STAI) and FCQ to measure the game’s impact on phobia symptom changes. The STAI questionnaire comprises 40 questions, measuring state (S-scale) and trait (T-scale) anxiety using a 4-point Likert scale. Only the S-scale was used in this study. The evaluation of state anxiety can be used for any situation with a time interval determined by a researcher or a clinical specialist. Mahram developed the Persian version of the STAI, and its internal consistency was confirmed for the S- and T-scales (Cronbach α of .91 and .90, respectively) [40]. Another Iranian study also reported high reliability for the S- and T-scales with Cronbach α values of .93 and .90, respectively [41]. The FCQ questionnaire was derived from the Fear of Spiders Questionnaire (FSQ) to assess cat phobia, with all instances of the word “spider” replaced by “cat.” Furthermore, the question format was adjusted to suit the assessment of the cat phobia. The FSQ is an 18-item tool scored on a 7-point Likert-type scale to measure the level of spider phobia, yielding a total score ranging from 18 to 126. The FSQ demonstrates excellent internal consistency with Cronbach α ranging from .88 to .97 [42,43] and good test-retest reliability [42]. The FSQ has been used in previous studies for various phobias such as cockroaches [7,44,45], rats [46], and snakes [47].

After the games, each group of participants was instructed to play 2 levels of the game as the opposite group did. They were then asked to answer the following questions: (1) Which experiences do you prefer? (2) Which experiences had more novelty and were more attractive to you? (3) Which experience was more effective for improving your problem?

#### Patients’ Preferences About the Designed Treatment

To gather patients’ opinions on the implemented treatment, an adapted preference questionnaire [48] was used. This 6-item questionnaire focused on patients’ preferences regarding the types of cat models, their behaviors, sounds, and sizes. For example, the questions related to cat models are as follows: (1) If you could choose among the cat models, which one would you prefer? (2) Which cat model do you think would be more effective in helping you overcome your problem? (3) Which cat model do you find more logical for aiding in your progress? (4) Which of these cat models do you perceive as more aversive? (5) Which cat model would you recommend to a friend facing the same problem? (6) Are there any cat models missing in the game?

#### Heuristics Evaluations

The playability and usability aspects of the tool were examined through heuristic evaluations designed as semistructured interviews to optimize its performance and enhance usability. Participants completed a 5-Likert questionnaire covering user interfaces, VR experiences, BF, and game playability. Participants had the opportunity to provide additional comments. The evaluations incorporated 44 heuristics from studies [49-52]. We used Nielsen heuristics [49,50] to assess the interfaces, along with modified Nielsen principles for VR platforms [51]. In terms of game playability, a comprehensive evaluation was necessary to assess additional features, including gameplay, story, and mechanics, which went beyond simple interface usability evaluation [52]. We used the heuristic principles of playability introduced in [52], which carefully examine the various components of a game in terms of playability and enjoyment for the player, encompassing gameplay, mechanics, usability, and game story. In this study, we used the first 3 heuristics from this set.

#### Statistical Analysis

We evaluated the differences in subjective ratings of intrinsic motivation and levels of anxiety using ANOVA: 2-factor with replication of the total score, with session number as the time factor and task variant (the tool with and without BF) as the between-subjects factor. In addition, we used 1-way ANOVA with task variant as the between-subjects factor to investigate the effects of the tool on mitigating phobia symptoms. For analyzing the semistructured interviews, mean and SD scores were used.

## Results

### Participants

Of the 17 participants, 7 were excluded for (1) heart disease (n=1); (2) vision or balance problems (n=1; participants with VR-induced dizziness and severe nausea); (3) pregnancy (n=1); (4) personality disorders (n=1); and (5) fear of cats in specific situations (n=3; one was afraid of direct eye contact with cats, whereas 2 others were scared of black cats). Among the 10 included participants (Table 1), 1 individual had 2 other phobias: fear of public toilets (paruresis) and birds (ornithophobia), especially their beaks and legs. Another participant displayed general phobia of animals; even touching chicks elicited an electric shock response. In addition, the sight of cats, dogs (cynophobia) especially when they bark, and foxes caused annoyance and discomfort for her. Interestingly, she was more afraid of kittens than fully grown cats. Another patient had cynophobia and ailurophobia. Finally, 1 participant had a phobia of space and galaxies (to the extent of avoiding space-themed movies) as well as chicks phobia and ornithophobia stating, “I am even afraid of a bird in a cage that might come out and harm me*.*” This participant also avoided going to the park because of the fear of the animals. Given the prevalence of individuals experiencing multiple phobias, particularly fears related to various animals (zoophobia), such as cats, spiders, snakes, and dogs, it is crucial to explore the possibility of modifying the game to effectively address multiple types of phobias. The park environment appears to be conducive to addressing various animal phobias and specific phobias such as paruresis. Accessing 10 participants was hindered by the temporary filtering of Instagram in our country. In addition, 2 individuals declined to participate, expressing shyness and concerns about others noticing their phobia. Our observations suggest that men with ailurophobia conceal their fear more frequently. Notably, ailurophobia predominantly affected women, as 90% (9/10) of our participants were women (Table 1). Ailurophobia began in 70% (7/10) of the participants during childhood and 30% (3/10) during adolescence. The minimum and maximum ages of onset of phobia in the samples were 5 and 18 years, respectively. Regrettably, animal phobias in our country, particularly cat phobia, have been largely overlooked, leading individuals to live for many years in a completely curable condition without seeking treatment. Innovative and early interventions, for example, our tool, could treat patients from childhood when anxiety starts and reduce the negative impact of untreated phobias. There is a pressing need for screening and diagnostic games as a primary step, followed by therapeutic games. The main cause of participants’ phobia stemmed from an unexpected childhood encounter with a cat.

**Table 1:** Participants’ characteristics.

Characteristics			Non-BF^a^		BF
Age (years), mean (SD)			24 (7.31)		33.5 (7.16)
Female, n (%)			5 (100)		4 (80)
Video game playing hours per week, mean (SD)			6 (8.52)		1.5 (3.08)
Median level of education			Diploma degree		Master’s degree
Years living with cat phobia, mean (SD)			15.6 (5.68)		21.6 (12.01)
Married, n (%)			2 (40)		2 (40)
**The onset of phobia, n (%)**					
	Childhood	4 (80)		3 (60)	
	Adolescence	1 (20)		2 (40)	
	Youth	0 (0)		0 (0)	
	Adulthood	0 (0)		0 (0)	
Age of onset of phobia (years), range			9-13		5-18

^a^BF: biofeedback.

### Possible Positive Effects of the Gamified VRET Augmented With BF

#### Effect 1: Intrinsic Motivation

The average intrinsic motivation of the groups indicated better results for the BF group across all 4 mandatory games with 49 scores (the sum of motivation scores for BF vs non-BF in the first to fourth sessions were: 182 vs 174, 178 vs 169, 191 vs 160, and 182 vs 181). However, the results (*P* value [groups]=.15>.05=∝ and *F*_1,3_=2.165) indicate no statistically significant difference. The analysis used a 2-factor ANOVA with replication.

On the basis of the results (*P* value [sessions]=.91>.05=∝; *F*_3,3_=0.171), we can conclude that there were no significant differences in the effectiveness of the groups across the different sessions.

There were no significant differences in the interaction between groups and sessions (*P* value [interactions]=.61>.05=∝ and *F*_3,3_=0.609).

Of the 5 participants in the non-BF group, 4 played 2 levels using BF. Two of them chose each game version, whereas the other 2 preferred the BF version exclusively.

Overall, BF had a greater effect on motivating patients. With greater efforts to leverage its potential within the game, the positive impact on motivation can be substantially enhanced. Nevertheless, it is essential to note that the non-BF version fosters motivation by incorporating 2 vital motivational elements: gamification and VR.

As participants enter new and especially challenging stages, their internal motivation to play tends to decrease, whereas their anxiety increases. However, with repeated attempts at this stage, motivation gradually increased, and anxiety levels tended to decrease.

#### Effect 2: Simulating Fearful Situations

The non-BF group had, on average, 40 points higher anxiety scores across all 4 rounds of the forced games compared with the BF group (the sum of anxiety scores for BF vs non-BF in the first to fourth sessions were: 11 vs 33, 22 vs 34, 29 vs 34, and 27 vs 28). There was a statistically significant difference between the 2 groups (*P*=.009<.05=∝ and *F*_1,3_=7.805). The total anxiety score for the non-BF group was 129, whereas that for the other group was 89, indicating the beneficial role of BF in anxiety control. This finding also suggests that using BF could potentially reduce the need for a therapist’s presence. Caution is advised when interpreting these data, as it may be influenced by individuals with severe phobias. The crucial point is that both game variants can evoke anxiety, as they simulate fearful situations. During the games, 5 participants (4 without BF and 1 with BF) experienced extreme stress, necessitating temporary pauses to help them calm down. One participant even reported an increase in blinking frequency when feeling nervous while playing the game.

The *P* value (sessions)=.32>.05=∝ and *F*_3,3_=1.204, indicating no significant differences in the effectiveness of the groups across different sessions. Many participants experienced anxiety even before the games began, which significantly impacted their anxiety levels during training (picture step). One participant even mistook pictures of cats in the training as real cats because of high tension. In addition, 6 participants (4 without BF and 2 with BF) responded to the cat pictures. On the basis of the data and participant feedback, the order of increasing anxiety levels followed the sequence of stages, starting from the trial game and progressing through the forced games in the following order: fantasy, low-poly, and high-poly cats. Similarly, the normalization of cats occurred in the following order: fantasy cats, pictures of cats, low-poly cats, and high-poly cats. For instance, anxiety levels increased as the number of cats increased. No significant differences in interaction between groups and sessions were observed (*P* value [interactions]=.20>.05=∝ and *F*_3,3_=1.652).

#### Effect 3: Controlling Stressful Circumstances, Eliminating Therapists’ Presence, and Mitigating Catastrophic Thoughts

Most participants about the positive signs of reducing their catastrophic thoughts expressed that encountering cats had started to feel somewhat normal. They noted that with continued play, their irrational fears could be replaced with more rational ones, and these positive changes could extend beyond the game to real-life environments. One participant shared, “Before playing the game, I couldn’t even look at cats’ stickers or images, and I used to throw my toy cat out of my room window into the street*.”* Another participant expressed*, “*Encountering fantasy cats in small numbers has become normal for me, and I believe that over time, my fear of other types of cats will decrease*.*” Follow-up data are required to verify the lasting impact of these positive changes.

A total of six noteworthy comments on the elimination of therapists using BF were suggested: (1) after a few sessions, the game can be played independently without therapists; (2) the treatment process can be shortened; (3) patients with milder phobias can benefit from playing without therapists. Otherwise, therapists’ support is necessary during the initial sessions; (4) the game is more beneficial for therapists, offering a controlled environment free of danger; (5) combining virtual and face-to-face treatments is recommended, starting with the game to prepare patients for real-life cat encounters; and (6) BF cannot provide the psychological support therapists offer. One participant, Fatemeh, repeatedly reassured herself during gameplay, saying, “Fatemeh, it’s just a cat, it’s nothing, keep calm.” The necessity of a virtual therapist to provide reassurance and guidance during moments of severe anxiety was evident. Participants either managed to calm themselves or received assistance from us. At times, we had to explain the unlocked stage scenarios to convince the participants to proceed with the remaining games.

To enhance the effectiveness, some participants suggested that the game should display their effort by showing the minimum and maximum HR and the time taken to complete a level. In addition, 2 positive comments regarding HR were as follows: “I noticed that my fear is higher before encountering cats, but my heart rate decreases when I face them” and “Before playing, I believed my fear of cats was overwhelming, but the game helped me realize it wasn’t as intense as I thought.”

#### Effect 4: Preliminary Effects on Ailurophobia Treatment

Using ANOVA single factor, we could not detect a difference between the groups (*F*_1,8_=0.073, and *P* value=.79>.05=∝). The S-scale scores worsened by 50 and 33 points in the non-BF and BF groups, respectively (Table 2). Both variants induced anxiety, but the BF group showed lower anxiety levels, suggesting that BF was more effective in reducing stress.

No significant difference between the groups was detected (*P* value=.63>.05=∝, and *F*_1,8_=0.256). The non-BF group improved by 67 points in the FCQ scores, whereas the BF group worsened by 42 points (Table 3).

The significant difference in scores can be attributed to one participant in the non-BF group who initially experienced high anxiety before and during the game. However, as she played more games, her scores on the S-scale (64-28) and FCQ (119-13) decreased dramatically. She mentioned that she used to be greatly bothered by cats being near her or hearing their voices, but after playing the game, she felt less anxious. The constant presence of cats in the game and being able to hear their voices helped her overcome her fears. It is noteworthy that this participant played the game more than all other players, completing 10 levels, including the training stage. In the last 3 stages, the participants specified an anxiety level of 1 out of 10. Initially, we considered this participant’s data as an outlier, but because of the high number of games played, we retained her data. This observation clearly indicates that playing the game more frequently helps to normalize interactions with cats. Her anxiety scores (of 10) for playing 9 levels of the game were (the data related to training was excluded for all participants): 10, 10, 8, 3, 3, 2, 1, 1, 1. By replacing her score with a typical number, we obtained more reasonable scores. The non-BF and BF scores worsened by 9 and 42, respectively. Both game versions induced similar anxiety levels in participants. Some of the participants experienced symptom improvement. To assess the initial positive signs of phobia treatment using the FCQ, we should wait until the completion of 10 game stages on average. All participants completed this questionnaire shortly after the games (within a maximum of 10 minutes), and the effects of anxiety caused by fear were still evident. We had to reassure them that the game was not very scary and that the unpredictable event they feared would not happen in the next level, as 4 participants experienced extreme anxiety. These participants took longer breaks between the phases or temporarily stopped playing the game. This anxiety could adversely affect their grades. In addition, approximately 80% (8/10) of the participants mentioned that playing the game more often helped them become accustomed to seeing cats.

All participants expressed a preference for the gamified VRET with BF, stating that the experience was more novel and perceived as more effective in reducing fear.

**Table 2:** Pretest and posttest scores of S-scales.

Groups	Pretest	Posttest	Difference
Non-BF^a^ (control)	39	61	−22
Non-BF	64	28	36
Non-BF	38	67	−29
Non-BF	46	67	−21
Non-BF	33	47	−14
BF (experimental)	39	45	−6
BF	29	28	1
BF	42	65	−23
BF	39	37	2
BF	34	41	−7

^a^BF: biofeedback.

**Table 3:** Pretest and posttest scores of Fear of Cats Questionnaire.

Groups	Pretest	Posttest	Difference
Non-BF^a^ (control)	71	100	−29
Non-BF	119	13	106
Non-BF	82	94	−12
Non-BF	112	92	20
Non-BF	101	119	−18
BF (experimental)	84	97	−13
BF	70	82	−12
BF	76	104	−28
BF	89	85	4
BF	89	82	7

^a^BF: biofeedback.

### Patients’ Preferences About the Designed Treatment

Most participants expressed that the game had a positive therapeutic impact and was capable of normalizing their interactions with cats. In total, 2 participants played the game 7 and 10 times and reported significant changes in their perception of cat-related fears. They shared that their perceptions of cat fear transformed, and encountering cats felt normal. Moreover, they believed that this effect could be extended to real-life situations. One participant shared, “I used to feel uneasy when cats were nearby, and the sound of cats was distressing for me. But now, as cats are consistently present in the game, and the sound of cats is played during the gameplay, being close to cats and hearing their sounds has become completely normal for me.” In the last 2 stages, their anxiety levels were reduced to a rating of 1 of 10. Before playing, most participants anticipated that cats would appear in the forest and perch on the tree branches. They expected the paths where cats were located to have denser and more crowded areas, featuring an abundance of trees, wooden huts, and gazebos with cats nearby. One commented, “The space provided is too vast, and it could be made more intense to induce fear. It would be beneficial to create some narrower paths leading to a door where cats are positioned. This could instill more fear. Generally, the game’s paths are not challenging situations, and a darker environment could make the cat’s eyes more prominent.” These comments contradict most participants, who appreciated the game’s positive aspect of indirectly implementing treatment and displaying everyday interactions people have with cats. Incorporating various environments and cat behaviors could further normalize the interaction with cats from all angles. However, these changes must be introduced with caution to avoid reinforcing the perception that cats are scary. In addition, the suggested locations to be included in the game range from the park environment to urban settings, such as apartments, streets, alleys, markets, cafés, dark scenes, kitchens, and garbage cans.

Some participants preferred the fantasy cats, believing that they alone have the ability to normalize interactions with cats because they highlight the positive aspects of cats such as their beautiful eyes and portray them as attractive, safe, and less harmful. Designing different fantasy cats appears to be a reasonable way to encourage individuals. One participant said, “It bothers me that the cats’ heads are small and their tails are long. In contrast, fantasy models had big heads and short tails. In different game levels, placing fantasy cats next to other cats conveys the feeling that all cats are harmless. Starting with images of rough and fat cats and gradually increasing the number of cats, and transitioning them to real models, helped me realize that the initial stage’s image was merely in my mind and unreal. As the cats’ numbers increased, I discovered that they did not pose any harm.” These eye-opening opinions shed light on an overlooked aspect—the psychological impact of the game’s difficulty levels and cat types.

Preferring fantasy cats indirectly revealed that low- and high-poly cats mostly evoke fear. Most participants found these cats to be more rational. Increased aversion and avoidance were observed in places with more cat voices and presence. Longer sounds also intensified fear.

On the basis of these findings, it is suggested to gradually introduce sounds, starting from cats with no sounds to short and pleasant sounds and then to real single and multiple sounds. The maximum fear was near the boxes where the number and noise of the cats were higher. Although this arrangement was found to be effective and logical in normalizing interactions with cats, high fear levels may have led some participants to avoid playing altogether. One participant preferred orderly and grouped cats for a calmer experience, whereas disorderly placement near the box increased fear. These reasons highlight the significance of using fantasy cats. Most participants found the size of the cats were found to be suitable. However, larger cat sizes, such as pictures of striped cats and low-poly cats, increased anxiety. The picture level, considered the easiest, induced anxiety and fear in most participants (6 of 10). Concerning cats’ behavior, most preferred nonreactive cats, such as fantasy cats that simply look at the sky in a cartoony manner; cats sitting and grooming themselves; or cats moving along the path without any reaction. Most participants disliked black cats waving their hands or white cream cats turning and staring at the player.

Most participants expressed the need for the game’s cat designs to closely resemble real-world cats. The following cats were not used based on their comments:

- Spotted (mainly black and white) and gray-striped cats, which are abundant in Iran.
- Kittens: Participants made three points: (1) kittens may not have a significant therapeutic effect, but they enhance the game’s appeal and create a more lifelike environment; (2) the treasure finder can be replaced with a fantasy kitten, allowing for a more captivating display of less favored features of cats, such as their nails, tail, and head. Moreover, their beautiful eyes can be showcased as larger; and (3) the option of raising a kitten in the game.
- Fierce-looking cats with grabbing capabilities: adding them requires expert opinions. Although statistics on cat grabbing are limited, the actual occurrence is likely to be minimal. People’s intense fears may exaggerate this concern.
- A mother cat breastfeeding her babies for a heartwarming and motherly touch.
- Sphynx cats: despite being rare in Iran, could enhance realism and normalize fear of diverse cat breeds.
- Fat or fluffy cats resembling a doll-like appearance.
- Placing cats amidst the greens and bushes along the paths.
- Injured (eg, cats with one eye or leg) or lifeless cats.
- Sudden movements of cats (eg, cats leaping out of trash cans): mentioned by most participants.
- Feeding cats: some participants did not agree with implementing this feature.

In conclusion, the game layout and models were considered logical by most of the participants. They stressed that fighting with cats in the game could worsen their fear, making a clear distinction between a therapeutic game and one designed solely for entertainment purposes. This opinion is in agreement with the clinical expert (the fourth author) who emphasized that the games for treating animal phobias should avoid action and fighting scenarios. For example, reducing the fear of cockroaches using scenarios where they stomp on or kill them may adversely affect.

### Heuristics Evaluations

As presented in Tables 4-7, of the 44 heuristics adapted from the Nielsen user interface, VR, and playability, an impressive 41 principles obtained scores of 62% or higher, underscoring the tool’s potential as a therapeutic product. Moreover, it enhances patient adherence to the treatment process.

Overall, 90% (9/10) of the participants found learning to play the game remarkably easy, particularly with the convenience of using just one button under VR glasses, which proved beneficial for those with mobility disabilities. Two suggestions emerged concerning in-game movement: (1) incorporating a back button and (2) movement through walking, potentially achieved with motion-sensing devices. However, careful consideration is necessary to ensure that they positively impact the player experience. Some individuals may prefer a less cumbersome setup. To enhance experience, it is crucial to incorporate a tutorial in a video or audio format for first-time users by introducing relaxation techniques to manage panic situations. Many participants required clarification that frightful situations would not occur at the subsequent levels. Providing detailed descriptions of new levels, including information about cats’ types and behaviors, prevents players from creating self-made stories about cat attacks. Moreover, to improve clarity, players needed clearer instructions after opening each box, signaling that they should open 4 boxes per session. Although a ribbon in the corner of the screen displays the number of opened boxes, it does not adequately alert the players to this requirement. Among the VR principles, the navigation and orientation support principle excelled at 82%, with patients being well-informed about their in-game position. Notably, approximately 80% (8/10) of patients experienced no dizziness during extended gameplay. To increase the level of engagement and therapeutic impact, introducing a punishment mechanism, such as reducing players’ points, could be beneficial. It might be worth reconsidering the features of allowing players to win the game without encountering cats. Game sounds and music received a relatively low score (51%), causing tension and unease, instead of promoting peace and happiness. The addition of soothing natural sounds was also suggested. In addition, consider a sound to indicate proximity to the box, reducing the need to check the bar constantly and improving the focus on gameplay. The game could benefit from a save and resume feature, especially during panic situations, allowing patients to take a moment to calm down. Some also raised concerns about the suitability of graphics for older adult audiences.

The principle of variety in the game’s paths and challenges stands out as one of the main gameplay principles. Although it obtained a relatively good score (68%), most participants said that after a few stages, the game became monotonous. Players quickly realized that cats only appear in certain sections of the roads and near treasure boxes. Certain adjustments were recommended to enhance the game’s appeal. Increasing the spacing between trees and raising their height can create a more immersive environment. Adding colorful elements such as flowers, toys, water views, and a gazebo in the park will infuse vibrancy into the game. In general, elevating the game’s attractiveness can be achieved by introducing a greater sense of adventure without relying on unrealistic fears. One participant suggested that instead of having the treasure box as the game’s goal, it could be placed in various locations within the forest, each rewarding the player with different prizes, such as food. Another suggestion was to replace the guide bar, which received positive feedback from the participants, with a map that indicated the approximate distance to the target. In addition, the introduction of a captivating and fantastical cat character instead of the current bar was recommended. In total, 2 participants pointed out that displaying HR in the corner might be somewhat distracting. It was suggested to show HR only when it was high or to remind players to reduce stress using a heartbeat’s sound.

**Table 4:** Results of the questionnaire designed based on [49-51] for evaluating user interfaces and virtual reality apps, respectively (Tables S1-S4 of Multimedia Appendix 1 provides the noncompressed version of Tables 4-7 containing the list of questions).

Usability heuristic and question			Question, mean (SD)		Heuristic, mean (SD)		Heuristic overall percent, %
**1. Natural engagement**							
	Q1	3.5 (1.08)		3.7 (0.28)		74	
	Q2	3.9 (0.88)		N/A^a^		N/A	
**2. Compatibility with the user’s task and domain**							
	Q3	3.7 (0.95)		3.7 (0.3)		74	
	Q4	4 (1.25)		N/A		N/A	
	Q5	3.4 (1.17)		N/A		N/A	
**3. Natural expression of action**							
	Q6	3.5 (1.27)		3.2 (0.42)		64	
	Q7	2.9 (1.20)		N/A		N/A	
**4. Close coordination of action and representation**							
	Q8	3.5 (1.27)		3.6 (0.1)		72	
	Q9	3.6 (1.17)		N/A		N/A	
	Q10	3.7 (1.25)		N/A		N/A	
**5. Realistic feedback**							
	Q11	3.7 (1.4)		3.7 (1.4)		74	
**6. Faithful viewpoints**							
	Q12	3.6 (1.2)		3.6 (1.2)		72	
**7. Navigation and orientation support**							
	Q13	4.1 (1.2)		4.1 (1.2)		82	
**8. Visibility of system status**							
	Q14	3.7 (1.25)		3.63 (0.75)		72.5	
	Q15	3.4 (1.17)		N/A		N/A	
	Q16	3.7 (1.49)		N/A		N/A	
	Q17	3.7 (1.16)		N/A		N/A	
**9. Consistency and standards**							
	Q18	3.7 (1.3)		3.7 (1.3)		74	
**10. Error prevention**							
	Q19	3.7 (1.34)		3.5 (0.28)		70	
	Q20	3.3 (2)		N/A		N/A	
**11. Recognition rather than recall**							
	Q21	3.6 (1.2)		3.6 (1.2)		72	
**12. Flexibility and efficiency of use**							
	Q22	3.8 (1.0)		3.8 (1.0)		76	
**14. Help and documentation**							
	Q23	2.9 (1.4)		2.9 (1.4)		58	

^a^N/A: not applicable.

**Table 5:** Results of the questionnaire designed based on gameplay part of the playability heuristics [52].

Question		Question, mean (SD)	Heuristic, mean (SD)	Heuristic overall percent
**1. Player’s fatigue is minimized by varying activities and pacing during game play.**				
	Q1	3.4 (1.6)	3.4 (1.6)	68
**2. Provide consistency between the game elements and the overarching setting and story to suspend disbelief.**				
	Q2	3.4 (1.3)	3.4 (1.3)	68
**3. Provide clear goals, present overriding goal early as well as short-term goals throughout play.**				
	Q3	4.3 (1.1)	4.3 (1.1)	86
**4. There is an interesting and absorbing tutorial that mimics game play.**				
	Q4	4.5 (1.0)	4.1 (0.57)	90
	Q5	3.7 (0.9)	N/A^a^	N/A
**5. The game is enjoyable to replay.**				
	Q6	3.5 (0.7)	3.5 (0.7)	70
**6. Game play should be balanced with multiple ways to win.**				
	Q7	3.8 (1.0)	3.8 (1.0)	76
**7. Player is taught skills early that you expect the players to use later, or right before the new skill is needed.**				
	Q8	3.4 (1.5)	3.4 (1.5)	68
**8. Players discover the story as part of game play.**				
	Q9	4 (0.8)	4 (0.8)	80
**9. The game is fun for the Player first, the designer second and the computer third. That is, if the nonexpert player’s experience is not put first, excellent game mechanics and graphics programming triumphs are meaningless.**				
	Q10	3.9 (1.2)	3.9 (1.2)	78
**10. Player should not experience being penalized repetitively for the same failure.**				
	Q11	4.3 (0.7)	4.3 (0.7)	86
**11. Player’s should perceive a sense of control and impact onto the game world. The game world reacts to the player and remembers their passage through it. Changes the player makes in the game world are persistent and noticeable if they back-track to where they have been before.**				
	Q12	4.1 (1.0)	3.9 (0.28)	82
	Q13	3.7 (1.3)	N/A	N/A
**12. The game should give rewards that immerse the player more deeply in the game by increasing their capabilities (power-up), and expanding their ability to customize.**				
	Q14	3.5 (1.3)	3.7 (0.28)	70
	Q15	3.9 (1.0)	N/A	N/A
**13. Pace the game to apply pressure but not frustrate the player. Vary the difficulty level so that the player has greater challenge as they develop mastery. Easy to learn, hard to master.**				
	Q16	3.9 (1.2)	3.75 (0.21)	78
	Q17	3.6 (1.2)	N/A	N/A
**14. Challenges are positive game experiences, rather than a negative experience (results in their wanting to play more, rather than quitting).**				
	Q18	3.8 (1.1)	3.8 (1.1)	76

^a^N/A: not applicable.

**Table 6:** Results of the questionnaire designed based on mechanic part of the playability heuristics [52].

Question		Question, mean (SD)	Heuristic, mean (SD)	Heuristic overall percent
**1. Game should react in a consistent, challenging, and exciting way to the player’s actions (eg, appropriate music with the action).**				
	Q1	2.8 (1.6)	2.8 (1.6)	51
**2. Make effects of the AI^a^ clearly visible to the player by ensuring they are consistent with the player’s reasonable expectations of the AI actor.**				
	Q2	3.1 (0.9)	3.1 (0.9)	62.3
**3. A player should always be able to identify their score/status and goal in the game.**				
	Q3	4.4 (0.5)	4.3 (0.14)	86
	Q4	4.2 (0.9)	N/A^b^	N/A
**4. Mechanics/controller actions have consistently mapped and learnable responses.**				
	Q5	4.4 (1.1)	4.15 (0.35)	83
	Q6	3.9 (1.6)	N/A	N/A
**5. Shorten the learning curve by following the trends set by the gaming industry to meet user’s expectations.**				
	Q7	4.3 (1.3)	3.95 (0.49)	79
	Q8	3.6 (1.6)	N/A	N/A
**6. Controls should be intuitive, and mapped in a natural way; they should be customizable and default to industry standard settings.**				
	Q9	4.5 (1.0)	4.35 (0.21)	87
	Q10	4.2 (0.9)	N/A	N/A
**7. Player should be given controls that are basic enough to learn quickly yet expandable for advanced options.**				
	Q11	3.3 (1.3)	3.53 (0.32)	70.67
	Q12	3.4 (1.7)	N/A	N/A
	Q13	3.9 (1.4)	N/A	N/A

^a^AI: artificial intelligence.

^b^N/A: not applicable.

**Table 7:** Results of the questionnaire designed based on usability part of the playability heuristics [52].

Question		Question, mean (SD)	Heuristic, mean (SD)	Heuristic overall percent
**1. Provide immediate feedback for user actions.**				
	Q1	4.1 (1.5)	4.1 (1.5)	82
**2. The player can easily turn the game off and on, and be able to save games in different states.**				
	Q2	2.3 (1.3)	2.3 (1.3)	46
**3. The player experiences the user interface as consistent (in control, color, typography, and dialog design) but the gameplay is varied.**				
	Q3	3.7 (1.3)	3.35 (0.49)	67
	Q4	3 (1.2)	N/A^a^	N/A
**4. The player should experience the menu as a part of the game.**				
	Q5	3.4 (1.0)	3.65(0.35)	68
	Q6	3.9 (1.0)	N/A	N/A
**5. Sounds from the game provide meaningful feedback or stir a particular emotion.**				
	Q7	3.5 (1.2)	3.35 (0.35)	67
	Q8	3.2 (1.1)	N/A	N/A
	Q9	2.8 (1.6)	N/A	N/A
**6. Players do not need to use a manual to play the game.**				
	Q10	4 (0.9)	4 (0.9)	80
**7. Make the menu layers well organized and minimalist to the extent the menu options are intuitive.**				
	Q11	3.9 (1.6)	3.9 (1.6)	78
**8. Get the player involved quickly and easily with tutorials and/or progressive or adjustable difficulty levels.**				
	Q12	4.1 (1.3)	3.95 (0.21)	79
	Q13	3.8 (1.4)	N/A	N/A
**9. Art should be recognizable to the player, and speak to its function.**				
	Q14	3.7 (1.2)	3.65 (0.07)	73
	Q15	3.6 (0.7)	N/A	N/A

^a^N/A: not applicable.

## Discussion

### Principal Findings

We developed a gamified VRET augmented with BF to address ailurophobia. To our knowledge, no specialized research on ailurophobia treatment exists, either in Iran or internationally. Motivated by the high prevalence of ailurophobia and the lack of accessible gamified VR environments with BF, our main goal was to create and assess a smartphone-based VRET augmented with BF for animal phobia (cat phobia). We hypothesized that this tool would better motivate patients, manage stress, simulate fearful situations, treat phobia, and reduce therapists’ involvement compared with a gamified VRET. The tool was designed based on expert sessions in video games, gamification, cognitive, and psychology. The results indicate its positive impact on specified features. Of the 44 heuristics, 41 scored above 62%, showing the potential for phobia interventions and motivating patients for treatment. Although tested on only 10 participants for a short duration (up to 3 hours without follow-up sessions), the results were reliable. Extensive data and feedback collection have been used to evaluate various aspects of the tool. On average, after 10 sessions, initial signs of improvement were observed, with slight variations depending on individuals’ phobia levels. One intriguing finding was that most participants were content with the game’s indirect approach to normalize interaction with cats and its nonviolent nature. They emphasized that action or combat scenarios would reinforce unrealistic fears and validate their phobia. Another significant finding was the progression of normalization in dealing with cats, tolerating their behavior, and hearing their voices, which gradually became more challenging. Although the current game normalizes communication with cats and holds good appeal, most participants suggested improvements, such as adding a variety of cats that closely resemble real-world characteristics, including voices and behaviors, to further enhance the normalization process. In addition, most participants expressed satisfaction with the game’s easy movements and minimal learning curve. To enhance the experience, adding diversity and adventure while minimizing unrealistic violence was recommended. Moreover, during the evaluations, the participants strongly felt the need for a virtual therapist to provide calming guidance and support during moments of severe anxiety.

### Comparison With Prior Work

To our knowledge, no study has simultaneously used BF, VR, and gamification for the treatment of animal phobia. However, various studies have used VR and game concepts to address specific animal phobias, for example, spider phobia [12,32] and snake phobia [47]. Similar to these studies, our tool successfully induced anxiety and led to a reduction in fear levels, avoidance behaviors, and catastrophic thoughts related to phobias. In addition, it positively boosted their motivation for treatment adherence. Unlike previous studies, our unique feature was the initial evaluation, showing that participants preferred a gamified VRET with BF. It has proven to be more effective in reducing symptoms and increasing internal motivation. These findings align with recent reviews highlighting the significant anxiety-reducing benefits of combining VR and BF, along with the advantages in motivation, user experience, involvement, and attentional focus [53,54]. In contrast to our study, where more participants preferred interacting with safe stimuli such as fantasy cats, studies such as those by Dibbets and Schruers [55] and Pittig et al [56] reported that selecting riskier options led to a stronger decrease in self-reported spider fear and disgust, whereas safe choices increased these emotions. The differing outcomes could be attributed to the use of VR and 3D images. VRETs are widely recognized as an appealing treatment modality because of their perceived naturalness in the automated format. However, Albakri et al [57] suggested that augmented reality exposure therapies offer a better experience and increased realism by seamlessly integrating digital information into the real world rather than creating a completely new virtual environment. We plan to explore the implementation of our designed tool with augmented reality and compare the outcomes in future studies. Dibbets and Schruers [55] found that the number of spiders encountered did not correlate with declines in aversive feelings and avoidance behaviors. However, our study concluded that a higher number of stimuli were more effective in normalizing interactions with cats. In addition, we observed that the action and combat scenarios were not beneficial for individuals with phobias. Interestingly, snake phobia treatment in a nearly action genre format [47] lacks a rationale for its selection. Further research is required to determine and devise appropriate scenarios for individuals with phobias. Throughout this study, the need to conduct similar research was highlighted. It was not feasible to make precise comparisons with prior studies in every detail.

### Limitations

The initial study on treating ailurophobia using VRETs with gamification and BF had limitations, primarily a small number of respondents. A total of 10 potential participants were inaccessible after Instagram’s temporary filtering in our country. The sample was skewed toward educated participants in their twenties and thirties, indicating the need to include diverse educational backgrounds, children, adolescents, and older adults. Owing to time constraints, we did not use any statistical method to calculate the required sample size. The study by Mor et al [48] recommended a minimum of 20 participants in each study arm for feasibility pilot studies on treating flying phobia using 360° images. Certainly, a larger number of patients is needed in each arm for the primary assessments. One future work is to replicate the study quantitatively and more rigorously while also introducing another arm that uses standard and clinical exposure therapies, enabling us to evaluate the tool and showcase more applications. In addition, the small sample sizes prohibited us from examining dropout rates. The results are exploratory, and long-term effects remain unknown due to the lack of follow-up data. Only one self-rating scale, the FCQ, has been used to diagnose individuals with ailurophobia. However, it is advisable to supplement such questionnaires with a telephone or face-to-face diagnostic interview conducted by an expert clinician, typically lasting approximately 30 minutes [2,12,32]. These interviews not only boost diagnostic reliability but also enable descriptive analysis [2]. It is worth mentioning that the patients were initially asked to explain the origin and signs of their ailurophobia. Participants were randomly divided into groups; however, the equality of their stress levels was not considered. It appears that by preserving randomness, the stress levels of individuals in the study groups should be nearly equal. For example, if one group has 2 extreme cases, the other groups should also have 2 similar cases to ensure transparency and enhance the reliability of the results. Creating a real-world game proved challenging owing to the limitations of the smartphone platform. Although playability and system usability questionnaires were not rigorously assessed, they were designed based on popular usability scales, including Nielsen [49,50], VR [51], and playability [52] heuristic evaluations. Changes in the individual’s physiological status, particularly HR, influence their experience. Unfortunately, this feature could not be assessed in the BF arm owing to the small sample size. Understanding its effectiveness in high-tension situations and its role in reducing anxiety remains a top priority.

### Conclusions and Future Work

The gamified VRET incorporating BF for treating cat phobia could be effective and has the potential to evolve into a comprehensive tool. One way to enhance its utility is by expanding the variety of cat types and behaviors, simulating different environments where cats are commonly found, and boosting its appeal through increased adventure while avoiding the use of unrealistic fears. After modifying the tool and using more robust study designs with ample sample sizes, further investigation can explore how this tool can be used in treatments without the presence of a therapist or combined (virtual and real simulation of fear), both in clinics and remotely. The park environment has the potential to effectively treat various animal phobias and other specific phobias. Implementing a gradual progression of sound stimuli could improve the therapeutic process. Starting with serene and pleasant sounds and gradually advancing to more challenging and potentially distressing voices, like cats squealing (inspired by a participant’s recollection of hearing a cat giving birth) or their aggressive vocalizations during fights. The final suggestion is to add the possibility of interacting with cats during more challenging stages, thereby bridging the game environment with the real world.

## Appendix

Multimedia Appendix 1 Questionnaires were designed based on user interfaces, virtual reality, and playability heuristics, along with their results.

## Abbreviations

BF: biofeedback
FCQ: Fear of Cats Questionnaire
FSQ: Fear of Spiders Questionnaire
HR: heart rate
STAI: State-Trait Anxiety Inventory
VR: virtual reality
VRET: virtual reality exposure therapy

## Data Availability

The data presented in this study are available from the corresponding author upon request.
