# Supplementary material for: A Smartphone-Gamified Virtual Reality Exposure Therapy Augmented With Biofeedback for Ailurophobia: Development and Evaluation Study
Source: JMIR Serious Games. 2024 Mar 6;12:e34535. doi: 10.2196/34535 (PMC10955409; doi:10.2196/34535)
Supplement: Multimedia Appendix 1 [file games_v12i1e34535_app1.doc]

**Multimedia Appendix 1**

**Table S1. R**esults of the questionnaire designed based on [49,50] and [51] for evaluating user interfaces and VR applications, respectively

| **Usability heuristic** | **Question** | **Mean (SD)** | **Mean (SD)** | **Overall percent** |
| --- | --- | --- | --- | --- |
| 1. Natural engagement | 1-How to move and interact with the environment is subject to the rules of the real world. | 3.5 (1.08) | 3.7 (0.28) | 74% |
|  | 2- The game, by providing conditions similar to the real world, can simulate frightening stimuli in the real world. | 3.9 (0.88) |  |  |
| 2. Compatibility with the user’s task and domain | 3- The order of difficulty of the steps is presented in a logical way and according to the level of phobia of people. | 3.7 (0.95) | 3.7 (0.3) | 74% |
|  | 4- The degree of difficulty of the stages can be customized according to the degree of phobia of people. | 4 (1.25) |  |  |
|  | 5- The appearance of the virtual world and the scary elements in it can simulate scary stimuli in the real world. | 3.4 (1.17) |  |  |
| 3. Natural expression of action | 6- Movement tools and interaction with the virtual environment available to the user are consistent with physical interactions and movements in the real world. | 3.5 (1.27) | 3.2 (0.42) | 64% |
|  | 7- The game provides the possibility of interacting with the components of the virtual world, similar to the real world. | 2.9 (1.20) |  |  |
| 4.Close coordination of action and representation | 8- During the game, feedback on movements and interactions and changes in the virtual world is provided quickly. | 3.5 (1.27) | 3.6 (0.1) | 72% |
|  | 9-While playing the game, the possibility of dizziness caused by the mismatch between the virtual and real world was minimal. | 3.6 (1.17) |  |  |
|  | 10- Playing the game for a long time does not cause dizziness. | 3.7 (1.25) |  |  |
| 5. Realistic feedback | 11- The reaction and behavior of the elements and stimuli in the game is close to the real world and can simulate the fear in the real situation. | 3.7 (1.4) | 3.7 (1.4) | 74% |
| 6. Faithful viewpoints | 12- The change in the image is rendered well according to the head movement and according to the rules of the real world. | 3.6 (1.2) | 3.6 (1.2) | 72% |
| 7. Navigation and orientation support | 13-The user is informed of her/ his current position on the playing field. | 4.1 (1.2) | 4.1 (1.2) | 82% |
| 8. Visibility of system status | 14- This game informs the user about what is happening during the game. | 3.7 (1.25) | 3.63 (0.75) | 72.5 % |
|  | 15- After the end of each stage, the feedback received by the user shows the beginning of the next stage well. | 3.4 (1.17) |  |  |
|  | 16- While playing the game, the user is aware of her/ his progress in the game. | 3.7 (1.49) |  |  |
|  | 17- By providing suitable and logical conditions, the game indirectly determines the path to reach the goal at each stage. | 3.7 (1.16) |  |  |
| 9.Consistency and standards | 18- The rules of interaction with the virtual world are consistent throughout the game and similar situations have the same interaction rules. | 3.7 (1.3) | 3.7 (1.3) | 74% |
| 10. Error prevention | 19- During the process of playing the game, errors never or rarely occur. | 3.7 (1.34) | 3.5 (0.28) | 70% |
|  | 20- If an error occurs in any part of the game, the error and its cause will be notified to the user. | 3.3 (2) |  |  |
| 11.Recognition rather than recall | 21- The connection between the behavior of the elements in the virtual world and the behavior they show makes it easy to recognize how to interact with them and minimizes the memory load and the need to remember the background of the elements. | 3.6 (1.2) | 3.6 (1.2) | 72% |
| 12. Flexibility and efficiency of use | 22- Considering that the intensity of phobia is different in people and people may not react to scary stimuli with less intensity, the game allows the user to adjust the stimuli based on the intensity of the phobia. | 3.8 (1.0) | 3.8 (1.0) | 76% |
| 14. Help and documentation | 23-A comprehensive guide and instruction for using the game is included in the game. | 2.9 (1.4) | 2.9 (1.4) | 58% |

**Table S2.** Results of the questionnaire designed based on playability heuristics [52], gameplay heuristics

| **#** | **Question** | **Mean (SD)** | **Mean (SD)** | **Overall percent** |
| --- | --- | --- | --- | --- |
| 1. Player’s fatigue is minimized by varying activities and pacing during game play. | 1-Variety in the paths and challenges in the game, can increase the excitement of the game? | 3.4 (1.6) | 3.4 (1.6) | 68% |
| 2.Provide consistency between the game elements and the overarching setting and story to suspend disbelief | 2- Unity has been created between the elements of the game, the main theme of the game and its story to increase believability and enjoyment in the player. | 3.4 (1.3) | 3.4 (1.3) | 68% |
| 3.Provide clear goals, present overriding goal early as well as short-term goals throughout play. | 3- Can the player figure out how to navigate the paths and reach the chests? | 4.3 (1.1) | 4.3 (1.1) | 86% |
| 4.There is an interesting and absorbing tutorial that mimics game play. | 4- How can the user easily learn to play? | 4.5 (1.0) | 4.1 (0.57) | 90% |
|  | 5- Can the game be used for all age groups with phobias? | 3.7 (0.9) |  |  |
| 5. The game is enjoyable to replay. | 6- Is the game attractive enough to repeat to continue treatment? | 3.5 (0.7) | 3.5 (0.7) | 70% |
| 6.Game play should be balanced with multiple ways to win. | 7- Can the player take paths with less scary stimuli to reach the end of each stage? | 3.8 (1.0) | 3.8 (1.0) | 76% |
| 7. Player is taught skills early that you expect the players to use later, or right before the new skill is needed. | 8-The skills needed by the player are taught to her/ his before she/ he needs them | 3.4 (1.5) | 3.4 (1.5) | 68% |
| 8.Players discover the story as part of game play. | 9- Can the story line match with the phobia during the game add to the appeal and effect of the game on the treatment of phobia? | 4 (0.8) | 4 (0.8) | 80% |
| 9.The game is fun for the Player first, the designer second and the computer third. That is, if the non-expert player’s experience isn’t put first, excellent game mechanics and graphics programming triumphs are meaningless. | 10- Has the game been able to provide the necessary simplicity for the use of different types of users with different levels of game experience while maintaining fun? | 3.9 (1.2) | 3.9 (1.2) | 78% |
| 10. Player should not experience being penalized repetitively for the same failure. | 11-The player should not feel that she/ he is punished every time for repeating a mistake | 4.3 (0.7) | 4.3 (0.7) | 86% |
| 11.Player’s should perceive a sense of control and impact onto the game world. The game world reacts to the player and remembers their passage through it. Changes the player makes in the game world are persistent and noticeable if they back-track to where they’ve been before. | 12- Movement in the game and interactions in it were under the control of the player? | 4.1 (1.0) | 3.9 (0.28) | 82% |
|  | 13- If the player returns to the boxes opened in the previous stages, will she/ he see the previous changes? | 3.7 (1.3) |  |  |
| 12. The game should give rewards that immerse the player more deeply in the game by increasing their capabilities (power-up), and expanding their ability to customize. | 14- Passing each stage can bring the player the feeling of overcoming her fear of scary elements. | 3.5 (1.3) | 3.7 (0.28) | 70% |
|  | 15- By passing each stage, the player can return to the previous points and find the strength to face the previous scary elements. | 3.9 (1.0) |  |  |
| 13. Pace the game to apply pressure but not frustrate the player. Vary the difficulty level so that the player has greater challenge as they develop mastery. Easy to learn, hard to master | 16- The intensity of the scary stimuli increases slowly during the game and does not make the player tired of the game. | 3.9 (1.2) | 3.75 (0.21) | 78% |
|  | 17- The role of the smart watch is noticeable in the difficulty of the stages and is consistent with the framework of the stages. | 3.6 (1.2) |  |  |
| 14. Challenges are positive game experiences, rather than a negative experience (results in their wanting to play more, rather than quitting). | 18- Does encountering scary elements in the game prevent the player from continuing the game? | 3.8 (1.1) | 3.8 (1.1) | 76% |

**Table S3.** Results of the questionnaire designed based on playability heuristics [52], mechanic heuristics

| **#** | **Question** | **Mean (SD)** | **Mean (SD)** | **Overall percent** |
| --- | --- | --- | --- | --- |
| 1. Game should react in a consistent, challenging, and exciting way to the player’s actions (e.g., appropriate music with the action). | 1- Can the sounds and music of the game be helpful in reducing the player's anxiety in tense moments for her/ his? | 2.8 (1.6) | 2.8 (1.6) | 51% |
| 2. Make effects of the Artificial Intelligence (AI) clearly visible to the player by ensuring they are consistent with the player’s reasonable expectations of the AI actor. | 2- The behavior of the scary elements in the game has the necessary believability and is similar to the elements Scary in the real world? | 3.1 (0.9) | 3.1 (0.9) | 62.3% |
| 3. A player should always be able to identify their score/status and goal in the game. | 3- Does the player know her position and score in the game? | 4.4 (0.5) | 4.3 (0.14) | 86% |
|  | 4- Being aware of the physiological state at every moment of the game can help to make the game fun and effective? | 4.2 (0.9) |  |  |
| 4. Mechanics/controller actions have consistently mapped and learnable responses. | 5- How to use and apply the game handle is simple and learnable? | 4.4 (1.1) | 4.15 (0.35) | 83% |
|  | 6- Is the use of a smartwatch in the game and its effect on the opening of chests understandable and learnable for the player? | 3.9 (1.6) |  |  |
| 5. Shorten the learning curve by following the trends set by the gaming industry to meet user’s expectations. | 7-The game does not offer unusual performances or mechanics that are difficult to learn. | 4.3 (1.3) | 3.95 (0.49) | 79% |
|  | 8- The way to use the smart watch in the game is according to the latest game making features and it will not be difficult to learn to interact with it. | 3.6 (1.6) |  |  |
| 6. Controls should be intuitive, and mapped in a natural way; they should be customizable and default to industry standard settings. | 9- How to use the game handle to move in the game environment, is it implemented in a natural way and according to the default of the game industry? | 4.5 (1.0) | 4.35 (0.21) | 87% |
|  | 10- The use of a game handle did not keep the player from being more immersed in the game. | 4.2 (0.9) |  |  |
| 7. Player should be given controls that are basic enough to learn quickly yet expandable for advanced options. | 11- Is it possible to change and personalize the game handle? | 3.3 (1.3) | 3.53 (0.32) | 70.67 |
|  | 12- Is it possible to set the heart rate threshold for each user? | 3.4 (1.7) |  |  |
|  | 13- Appropriate heart rate threshold for each person can increase the fun and effectiveness of the game to improve the player's phobia? | 3.9 (1.4) |  |  |

**Table S4.** Results of the questionnaire designed based on playability heuristics [52], usability heuristics

| **#** | **Question** | **Mean (SD)** | **Mean (SD)** | **Overall percent** |
| --- | --- | --- | --- | --- |
| 1. Provide immediate feedback for user actions. | 1- With the change in the physiological state of the person and the change in heart rate, will the user be informed of this change quickly? | 4.1 (1.5) | 4.1 (1.5) | 82% |
| 2. The Player can easily turn the game off and on, and be able to save games in different states. | 2- Is it possible to save the state of the game to resume it at another time? | 2.3 (1.3) | 2.3 (1.3) | 46% |
| 3. The Player experiences the user interface as consistent (in control, color, typography, and dialog design) but the gameplay is varied. | 3- Increasing the scary stimuli and changing their behavior according to each stage, can add to the variety in the gameplay and make the game more interesting. | 3.7 (1.3) | 3.35  (0.49) | 67% |
|  | 4- Is the appearance of the user interface consistent in all parts of the game and is it attractive enough? | 3 (1.2) |  |  |
| 4. The Player should experience the menu as a part of the game. | 5- Is the user interface in this game consistent with the general atmosphere of the game? | 3.4 (1.0) | 3.65  (.35%) | 68% |
|  | 6- Does the distance indicator from the treasure box in each stage and the heart rate indicator distract the player from the game process and scary stimuli? | 3.9 (1.0) |  |  |
| 5. Sounds from the game provide meaningful feedback or stir a particular emotion. | 7- Are the sounds of the game elements logical and believable and can they add to the panic in the player? | 3.5  (1.2) | 3.35 (0.35) | 67% |
|  | 8- Can changing the music according to the situation in this game help the player to reduce her anxiety? | 3.2 (1.1) |  |  |
|  | 9- Using the sound indicating the distance to the treasure box, can help the player get more involved in the game? | 2.8 (1.6) |  |  |
| 6. Players do not need to use a manual to play the game. | 10- The player does not need a guide to move in the environment and go through the steps. | 4 (0.9) | 4 (0.9) | 80% |
| 7. Make the menu layers well-organized and minimalist to the extent the menu options are intuitive. | 11- Nested windows in the main menu, implemented in a way that does not confuse the player? | 3.9 (1.6) | 3.9 (1.6) | 78% |
| 8. Get the player involved quickly and easily with tutorials and/or progressive or adjustable difficulty levels. | 12- Is it possible to set the difficulty of the steps for the user? | 4.1 (1.3) | 3.95 (0.21) | 79% |
|  | 13- Does this possibility help the player get more involved in the game? | 3.8 (1.4) |  |  |
| 9. Art should be recognizable to the player, and speak to its function. | 14-Are the models and elements in the game as well as its menus visually integrated? | 3.7 (1.2) | 3.65 (0.07) | 73% |
|  | 15- Are the visual elements of the game recognizable to the player and reminiscent of scary elements in the real world? | 3.6 (0.7) |  |  |
